# General practitioners’ views and preferences about quality improvement feedback in preventive care: a cross-sectional study in Switzerland and France

**DOI:** 10.1186/s13012-017-0623-7

**Published:** 2017-07-26

**Authors:** Paul Sebo, Hubert Maisonneuve, Jean-Pascal Fournier, Nicolas Senn, Dagmar M. Haller

**Affiliations:** 1 Primary Care Unit, Faculty of Medicine, University of Geneva, Geneva, Switzerland; 2 Department of General Practice, Faculty of Medicine, University of Nantes, Nantes, France; 3 Department of Ambulatory Care and Community Medicine, University of Lausanne, Lausanne, Switzerland; 4 Department of Community, Primary Care and Emergency Medicine and Department of Paediatrics, Geneva University Hospitals, Geneva, Switzerland

**Keywords:** Quality improvement, Feedback, Preventive care, Primary care

## Abstract

**Background:**

Feedback is widely used as a strategy to improve the quality of care in primary care settings. As part of a study conducted to explore the quality of preventive care, we investigated general practitioners’ (GPs) views on the usefulness of feedback and their preferences regarding how feedback is provided.

**Methods:**

This cross-sectional study was conducted in 2015 among randomly selected community-based GPs in two regions of Switzerland and France. GPs were asked to complete an anonymous questionnaire about how often they provided 12 measures of preventive care: blood pressure, weight and height measurements, screening for dyslipidemia, at-risk drinking (and advice to reduce for at-risk drinkers), smoking (and advice to stop for smokers), colon and prostate cancer, and influenza immunization for patients >65 years and at-risk patients. They were also asked to estimate the usefulness of a feedback regarding their preventive care practice, reason(s) for which a feedback could be useful, and finally, to state which type of feedback they would like to receive. Chi-square tests were used to compare frequencies. Multivariate logistic regression was used to identify factors associated with GPs considering feedback as useful.

**Results:**

Five hundred eighteen of 1100 GPs (47.1%) returned the questionnaire. They were predominantly men (62.5%) and most (40.1%) were aged between 55 and 64 years old. Overall, 44.3% stated that a feedback would be useful. Younger GPs and those carrying out more measures of preventive care were more likely to consider feedback useful. The two main reasons for being interested in feedback were to receive knowledge about the study results and to modify or improve practice. The two preferred feedback interventions were a brief report and a report with specific information regarding prevention best practice, whereas less than 1% would like to discuss the results face-to-face with the study investigators.

**Conclusions:**

These findings suggest that GPs have preferences regarding the types of feedback they would like to receive. Because the implementation of guidelines is highly related to the acceptance of feedback, we strongly encourage decision makers to take GPs’ preferences into account when developing strategies to implement guidelines, in order to improve the quality of primary care.

## Background

Much of preventive care is provided by general practitioners (GPs). Prevention is particularly important for the management of major modifiable risk factors, such as smoking, dyslipidemia, obesity, and high blood pressure [1]. Preventive care is an important part of GPs’ tasks which potentially contributes to reducing the burden of chronic diseases, such as heart diseases or cancers, and has the potential to decrease medical costs [2–5]. For example, measuring blood pressure within the practice is a noninvasive and inexpensive approach which may play a crucial role in the early detection (and treatment) of patients at high risk for the development of cardiovascular diseases.

Evidence-based guidelines for preventive care have been developed in different countries during the last decades [6, 7]. In Switzerland, a national program named EviPrev was recently launched to develop local guidelines, but these recommendations have not yet been implemented in clinical practice [8]. In France, the medical authorities decided to focus their guidelines on the management of diseases rather than on preventive care alone [9]. Though some aspects of preventive care are addressed in disease-specific guidelines, French GPs meet more difficulties accessing national preventive recommendations [9].

Monitoring how preventive care is delivered by GPs is essential to achieve a high quality of care. The single development of guidelines has little or no impact if there is no adoption and transfer to daily practice. However, adequate implementation of guidelines is crucial because they can really change clinical practice and improve patient outcome [10, 11]. The difficulty of adoption and transfer was confirmed by several studies carried out in the USA, which showed that the rates of preventive care were suboptimal: overall, only one half of recommended preventives services were usually provided [12–15]. In contrast, we previously reported high adherence by Swiss and French GPs to most recommendations for prevention (>70%), though certain measures were less often provided (above all, annual influenza vaccination for at-risk patients <65 years old) [16]. We also showed that, compared to French GPs, those practicing in Switzerland tended to provide slightly more measures.

GPs experience difficulties adhering to guidelines because of organizational constraints (in particular lack of time), insufficient financial compensation for providing preventive care, lack of awareness or familiarity with the guidelines, absence of agreement between the various guidelines, and difficulties in applying some recommendations in daily practice [17–19].

Several strategies have been suggested to increase adherence to guidelines, in particular interventions aimed at GPs (feedback, educational meetings, reminders, financial incentives, organizational changes in the practice), and regulatory interventions [10, 11, 20, 21]. By contrast, the single dissemination of guidelines without other measures is insufficient to ensure adequate GP adherence [21].

Several authors have shown feedback to be useful to improve health care, including preventive care [20]. It is based on the belief that healthcare professionals improve their practice when they receive feedback following audit showing suboptimal performances [20]. Using behavior change theories, it is hypothesized that feedback may change GPs’ awareness and beliefs about current practice, change their perceived social norms, and/or may lead them to focus on sub-goals. Within a framework based on control theory, GPs’ self-assessment of clinical performance and targets following audit and feedback serve to mobilize their intentions to improve their practice and adherence to guidelines [22]. A range of factors both specific to each physician (emotions, core values…) and linked to the environment (resources, workload…) determine whether physicians develop intentions to change in response to feedback and manage to put intentions into practice [23].

Several studies showed the effectiveness of feedback in preventive care. Whether GPs find feedback useful and above all, what type of feedback intervention they actually would like to receive is currently unknown [20, 24]. It is very important to take GPs’ preferences into account in order to increase the acceptability of feedback interventions and eventually increase guideline adherence in practice.

As part of a study that assessed the quality of preventive care in Western Switzerland (cantons of Geneva and Vaud) and in two French regions (Alsace, Pays de la Loire), we investigated the feasibility of a practice-based quality improvement feedback. We explored GP’s views on the usefulness of the feedback and their preferences for different feedback interventions.

## Methods

### Study site, study population, and sample size justification

We drew a random sample of 1100 community-based GPs (700 GPs practicing in Switzerland (Geneva and Vaud) and 400 in France (Alsace and Pays de la Loire)). The GPs were invited to participate in the study by post. Reminder messages (maximum twice by GP) were sent to non-responders. GPs who practiced complementary and alternative medicine were not eligible for the study. No other exclusion criteria were applied. The recruitment process, details about GPs’ selection, and sample size justification are described elsewhere [25].

### Data collection

Each randomly selected GP was contacted by post by a research assistant located in Geneva (for Switzerland) and by the local professional associations *Union Régionale des Professionnels de Santé Alsace* and *Pays de la Loire* (for France). GPs were informed about the aim of our study and the practical procedures for completing the questionnaire. The postal letters included a stamped return envelope. GPs were asked to fill out a questionnaire that included questions regarding socio-demographic characteristics (age group (<35, 35–44, 45–54, 55–64, >64), gender, location of the practice, certification, number of working days per week, number of working years in the current practice), and adherence to 12 preventive practices (see below).

These preventive practices were assessed with a 5-point Likert scale ranging from “never performed” to “always performed.” They were selected by consensus within the research team following a review of the literature. Ten preventive practices were included in the study because they had also been selected by previous authors: blood pressure, weight and height measurements, as well as screening for at-risk drinking (and advice to decrease drinking), for smoking (and advice to stop smoking), and for colon cancer, and finally, annual influenza immunization for patients >65 years and at-risk patients <65 years) [26]. We added two preventive measures to this list: cholesterol measurement because it is highly recommended and refraining from systematic screening for prostate cancer because several medical agencies recently recommended against systematic screening [9, 27, 28]. By contrast, we did not include diabetes screening because it was considered as targeted screening (i.e., limited to populations with particular risks factors such as obesity) or screening for breast cancer because this screening is often provided by gynecologists or in the context of screening programs [27, 29].

GPs were also asked to estimate the usefulness of a feedback regarding their preventive care practice (indispensable, very useful, rather useful, not very useful, useless) and to explain for which reason(s) they would find feedback useful (multiple answers allowed: to know GPs’ overall performance, to compare themselves with colleagues, to modify or improve their practice, to use regular feedback interventions to follow-up what is done in their practice, other reason). Finally, they were asked which type of feedback they would like (multiple answers allowed: a brief report (i.e., a report providing general information about GPs’ overall performance without individual and detailed results), a brief report and individual results, detailed results regarding their practice compared with the study results, a report and specific information regarding prevention best practice, a contact with the study investigators to discuss the results, a local quality circle meeting to discuss the results, other type of feedback). The selection of the feedback interventions was based on a consensus within the study team following a review of the literature [10, 11, 19, 21].

We pretested the questionnaire with seven GPs working in a primary care clinic (Centre Médical des Trois Chêne, Geneva, Switzerland) to ensure that the questionnaire was understandable and easy to complete. All collected data remained confidential throughout the study. It was assumed that tacit consent was given when a responder completed the questionnaire. No data was collected for the GPs who declined to participate.

### Statistical analyses

We computed the proportion of GPs delivering each of these preventive care measures, defined as the proportion of GPs scoring 4 or 5/5 (i.e., often or always performed) on the Likert scale, as well as the mean number of measures by GP. We also assessed, from the GPs’ perspective, the preferred feedback intervention(s) and the reason(s) why feedback interventions could be useful, according to GPs’ gender, age, country, and adherence to recommendations (<10 vs. ≥ 10 preventive care measures: 10 was the third quartile). Frequency tables and chi-square tests were used. Finally, a multivariate logistic regression was used to investigate which were the main factors (among the GPs’ characteristics and compliance with prevention measures) associated with the perception that feedback is indispensable or very useful. The final model was chosen using a stepwise procedure based on the Akaike information criterion. All analyses were performed with TIBCO Spotfire S+® 8.1 for Windows (TIBCO Software Corporation, Palo Alto, CA, USA) or R version 3.2.2 (R Foundation for Statistical Computing, Vienna, Austria).

## Results

Among the 1100 GPs who were contacted at random, 518 (47.1%) participated in the study. Table 1 presents their socio-demographic characteristics. They were predominantly men (62.5%) and practicing in Switzerland (70.6%). Most (40.1%) were aged between 55 and 64. The mean number of preventive care measures carried out by GPs was 9.0 (standard deviation 1.9). Overall, 44.3% estimated that feedback for preventive care recommendations would be indispensable or very useful (11.6% found feedback indispensable, 32.7% very useful, 36.7% rather useful, 10.0% not very useful, 9.0% useless).

**Table 1:** GPs’ socio-demographic characteristics (*N* = 518)

Characteristics		
	*n*/*N* ^a^	%
Gender		
Male	318/509	62.5
Female	191/509	37.5
Age group (years)		
<35	13/516	2.6
35–44	104/516	20.2
45–54	133/516	25.8
55–64	207/516	40.1
>64	59/516	11.4
Country		
France	163/518	39.4
Switzerland	355/518	70.6
	mean ± SD	
Mean number of half days worked per week	8.6 ± 2.3	
Number of working years in the current practice	18.7 ± 11.0	
Number of preventive care measures	9.0 ± 1.9	

^a^Numbers do not add to 518 because of missing data

Our sample appears to be relatively similar in age and gender to all community-based GPs practicing in Switzerland (professional organization of Swiss physicians, 2016: median age 54 years (vs. 54 years in our study); men 59% (vs. 61% in our study)) and France (Pays de Loire, 2013: median age 51 years (vs. 56 years in our study); men 57% (vs. 66% in our study) [30, 31].

Tables 2 and 3 show the distribution of the reasons for which GPs considered feedback useful and the types of feedback intervention that they would like to receive, overall and stratified by age group and gender, and by country of practice and number of prevention practices. Overall, the three main reasons given by GPs regarding the usefulness of feedback were to know GPs’ overall performance (50.6%), to modify or improve their practice (48.6%), and to compare her/himself with colleagues (42.1%); for younger GPs and women, the three main reasons for feedback were the same but in a different order. The two main types of feedback that GPs would like to receive were a brief report (54.4%) and a report with specific information regarding prevention best practice (37.8%); for younger GPs and women, the responses were equally balanced between these two types of feedback. Only 1.2% declared that they would like to discuss the results face-to-face with the study investigators. The findings according to country of practice and number of measures were similar, though Swiss GPs carrying out ≥10 measures were more likely to mention more than one reason for being interested in feedback.

**Table 2:** Reasons for finding feedback very useful or indispensable and preferred type of feedback stratified by GPs’ age category and gender

Characteristics^a^	Age <55 (*N* = 250)	Age ≥55 (*N* = 266)	*p* value	Male (*N* = 318)	Female (*N* = 191)	*p* value	Total (*N* = 518)
Reason for finding feedback very useful or indispensable (%)							
Know GPs’ overall performance	49.6	51.9	0.667	53.8	46.1	0.019	50.6
Comparison with my colleagues	45.2	39.1	0.189	38.4	48.2	0.038	42.1
Modify or improve my practice	58.8	39.5	<0.001	41.8	61.8	<0.001	48.6
Use regular feedback interventions to follow-up what is done in my practice	26.0	27.8	0.714	27.4	25.1	0.654	27.0
Preferred type of feedback (%)							
Brief report	51.6	56.8	0.276	59.7	47.1	0.007	54.4
Brief report and individual results	16.0	14.7	0.764	13.8	17.8	0.282	15.3
Detailed results regarding my practice compared with the study results	17.2	16.5	0.935	15.4	18.3	0.462	16.8
Report and specific information regarding prevention best practice	49.6	27.1	<0.001	32.7	47.1	0.002	37.8
Contact with the study investigators to discuss the results	0.8	1.5	0.738	1.6	0.5	0.524	1.2
Local quality circle meeting to discuss the results	8.8	6.8	0.485	6.6	9.9	0.235	7.7

^a^Numbers do not add to 518 because of missing data

**Table 3:** Reasons for finding feedback very useful or indispensable and preferred type of feedback stratified by country and number of prevention measures carried out by GPs

Characteristics	France (*N* = 163)	Switzerland (*N* = 355)	*p* value	Measures <10 (*N* = 281)	Measures ≥10 (*N* = 237)	*p* value
Reason for finding feedback very useful or indispensable (%)						
Know GPs’ overall performance	46.0	52.7	0.189	47.7	54.0	0.178
Comparison with my colleagues	38.0	43.9	0.242	40.2	44.3	0.395
Modify or improve my practice	44.2	50.7	0.198	45.2	52.7	0.104
Use regular feedback interventions to follow-up what is done in my practice	24.5	28.2	0.449	23.5	31.2	0.061
Preferred type of feedback (%)						
Brief report	57.1	53.2	0.475	52.3	57.0	0.332
Brief report and individual results	13.5	16.1	0.535	13.2	17.7	0.189
Detailed results regarding my practice compared with the study results	16.0	17.2	0.824	13.5	20.7	0.040
Report and specific information regarding prevention best practice	38.7	37.5	0.872	38.1	37.6	0.974
Contact with the study investigators to discuss the results	2.5	0.6	0.154	1.4	0.8	0.840
Local quality circle meeting to discuss the results	4.3	9.3	0.071	6.8	8.9	0.468

Table 4 shows the degree of usefulness of feedback (very useful or indispensable) according to GPs’ socio-demographic characteristics and adherence to prevention measures. The multivariate analysis showed that younger GPs and those carrying out ≥10 measures of preventive care were more likely to consider feedback very useful or indispensable.

**Table 4:** Univariate and adjusted associations of GPs’ characteristics and extent of preventive practice with their perceptions of the usefulness of feedback (very useful or indispensable vs. rather useful, little useful, or useless)

Characteristics				Multivariate		
	OR	95% CI	*p* value	Adjusted OR	Adjusted 95% CI	*p* value
Gender						
Male	1					
Female	1.372	0.955–1.973	0.086	^a^	^a^	^a^
Age group (years)						
<35	1			1		
35–44	0.337	0.073–1.554	0.013	0.286	0.049–1.661	0.018
45–54	0.232	0.051–1.057		0.222	0.039–1.261	
55–64	0.181	0.040–0.813		0.201	0.033–1.215	
>64	0.221	0.045–1.069		0.247	0.036–1.687	
Number of half days worked per week						
≤8	1					
>8	0.960	0.674–1.367	0.820	^a^	^a^	^a^
Number of working years in the current practice						
≤18	1					
>18	0.634	0.444–0.906	0.012	^a^	^a^	^a^
Location of the practice						
France	1					
Switzerland	1.399	0.956–2.046	0.081	^a^	^a^	^a^
Reported extent of preventive practice						
Commonly applies <10 preventive practices	1			1		
Commonly applies ≥10 preventive practices	1.865	1.310–2.655	<0.001	1.764	1.208–2.574	0.002

^a^Not selected in the adjusted stepwise selected multivariate model

## Discussion

### Summary of main findings

We showed that approximatively half of GPs found that feedback would be very useful for evaluating their clinical practice for preventive care and that younger GPs and those being more adherent to guidelines were more likely to consider feedback useful. We also showed that the two main reasons for feedback, as stated by GPs, were simply to have a view of GPs’ overall performance but also to modify or improve their practice. The two preferred feedback interventions were a brief report and a report with specific information regarding prevention best practice. Finally, we showed that very few GPs would like to discuss the results in local quality circle meetings and even less face-to-face with the study investigators.

### Comparison with the existing literature

Many authors showed that feedback may be useful to improve health care, including prevention [20, 32–35]. However, to our knowledge, few data existed on whether GPs were interested in receiving feedback and how they would like to receive it. Our results are consistent with those of a recent cross-sectional survey about Dutch GPs’ preferences for interventions to improve guideline adherence (*n* = 703 GPs) [21]. In this study, Lugtenberg et al. showed that GPs preferred interactive small group meetings (84%), but feedback (53%) was also rated positively. By contrast, financial incentives, the use of educational materials, or big group meetings were mentioned by less than one fourth of GPs.

Lugtenberg’s study was designed to identify GPs’ preferences regarding a wide range of interventions, whereas our study was restricted to feedback interventions only. This is probably the reason why interactive educational small group meetings seemed to be highly appreciated by Dutch GPs in Lugtenberg’s study, whereas they were not part of the preferred interventions selected in our study. In other words, these findings suggest that GPs may appreciate interactive meetings when they address and discuss their difficulties in applying guidelines in their daily practice but not when the objective of these meetings is to discuss their own performance.

The finding that very few GPs would like to discuss the results face-to-face with the investigators, or in local quality circle meetings, could be explained in the same way: fear or inconvenience of highlighting their (lack of) performance. We may also consider two alternative explanations: belief that the objective of the study investigators is to transfer knowledge to GPs in a unidirectional way in a top-down relationship, and time constraints [32]. GPs in our study preferred written formats of feedback (brief or detailed report sent to the participants) over verbal feedback (face-to-face contacts with the investigators or local quality circle meetings). Though there may be a perception that feedback delivered face-to-face is more efficient compared to written feedback, the evidence shows little or no difference between the two formats [20].

It is reassuring that the second main reason cited by GPs for receiving feedback was to improve clinical practice whereas the second preferred type of intervention was a report with specific information regarding best practice: this is precisely why feedback is recommended [20]. This finding, and the fact that half of GPs found feedback useful, tends to suggest that implementation of a practice-based quality improvement feedback is feasible and acceptable to a large number of GPs. This is particularly true if feedback is written and not transmitted orally. Indeed, our results suggest that limited acceptability may be expected for face-to-face discussion with clinicians.

That younger GPs and those being more adherent to guidelines were more likely to consider feedback useful suggests that they are probably more open to criticism and more receptive to feedback seen as a way of improving their practice. Younger GPs (maybe because they are in general less experienced clinicians and, as a result, have not yet established their style of care) seem to have higher adherence to guidelines [36–38]. For example, in a study assessing GPs’ attitudes to guidelines for elective surgical referral in England, it was shown that the odds of using guidelines decreased with increasing age, a 10-year increase in age being associated with halving odds of use [38]. By extension, it is hypothesized that younger GPs (and those being more adherent to guidelines) are also more receptive to feedback and therefore more likely to consider these interventions useful for improving their skills.

### Limitations

Only GPs practicing in Western Switzerland and two regions in France were invited to take part in the study, and only 47% of those who were contacted agreed to participate. Those who replied were likely to feel more concerned by the study question, which may have inflated the number of GPs who found feedback indispensable or very useful. However, response rates for surveys conducted among GPs are generally lower than surveys among other medical doctors, [39] and responses rates similar to ours are frequent in primary care research. As the answers were self-reported, our findings could partially be explained by the fact that responders may have a natural tendency to over-report positive, socially desirable behaviors (social desirability bias) [40]. In addition, our findings were obtained in a research context and may have been different if acquired in the context of a preventive guideline implementation project. As we did not collect any data on non-responders, we could not assess potential differences between responders and non-responders. However, our sample appears to be relatively representative in terms of age and gender of all community-based GPs practicing in Switzerland and France (data from Pays de la Loire). We believe that the risk of bias due to missing data is probably very small because there were only few missing data in our study. We used a stepwise selection procedure rather than a hypothesis-driven selection for the multivariate analysis of usefulness perceptions, which could lead to a model giving an over-optimistic impression despite the additional fitting error this may have added. Though many authors have shown that feedback may be useful to improve health care, including in prevention, we did not know to the best of our knowledge if GPs found feedback useful. Therefore, it would have been difficult to make theoretical hypotheses about the most likely predictors of usefulness perceptions to include in the model. Finally, after reviewing the literature, we decided to keep only five response options for the question assessing the reason(s) why GPs would find feedback useful, respectively seven options for the question about the type(s) of feedback they would like. These options seemed to be the most interesting, important, or pertinent to study in the context of primary care. We decided to limit these response options because with questions offering many possible response options, we were afraid that certain questions would have been completed incorrectly, for example at random. Though this restricted selection could result in a certain degree of information bias, we do not believe that this was in fact the case. Indeed, though “other reason” and “other type of feedback” were response options proposed to responders, they were never selected by GPs in our sample. In addition, no GP (*n* = 7) suggested in the pretest phase that we add other response options to the list.

### Implications for practice and policy

GPs are increasingly placed under pressure to improve the quality of care. While the aim of guidelines is precisely to achieve a high quality of care (by providing specific recommendations for daily practice), their implementation in practice is highly challenging.

Several authors studied barriers to guideline adherences, but little is known on how to overcome these barriers [17, 18, 21]. Several interventions have been suggested and among these, feedback is increasingly used in primary care [10, 11, 20, 21]. Unfortunately, GPs are not usually involved in the choice of the format of the intervention chosen by decision makers to improve their practice [21]. However, it is likely that GPs’ adherence to guidelines could be improved if their views were taken into account [10]. In this context, we believe that our findings could help decision makers when they develop strategies to implement guidelines in primary care.

Such interventions are probably more efficient if they are repeated periodically [20]. Quality control circles (QCC) using PDCA (plan-do-check-act) iterative methods could be used for quality improvement feedback in preventive care [41]. QCC are used in healthcare to increase the quality of medical practice by establishing objectives (plan), implementing a plan (do), studying the results and comparing against the expected results (check), and making adjustments if necessary (act) [41–44]. One of the main advantages of this method, when it is adequately carried out, is continuous monitoring, favoring sustained improvement. PDCA cycles can also be conducted by GPs themselves (potentially supported by a feedback system, e.g. a quality dashboard) without investigator involvement.

## Conclusion

Approximatively half of GPs found that feedback was useful for the evaluation of their preventive care practices, and they had clear preferences regarding the type of feedback they would like to receive. Because the implementation of guidelines is highly related to the acceptance of feedback, we strongly encourage decision makers to take GPs’ preferences into account when developing strategies to implement guidelines, in order to improve quality in primary care.

## Abbreviations

GPs: General practitioners
PDCA: Plan-do-check-act
QCC: Quality control circles
